# Pentaplex real‐time PCR for differential detection of *Yersinia pestis* and *Y*.* pseudotuberculosis* and application for testing fleas collected during plague epizootics

**DOI:** 10.1002/mbo3.1105

**Published:** 2020-08-12

**Authors:** Ying Bai, Vladimir Motin, Russell E. Enscore, Lynn Osikowicz, Maria Rosales Rizzo, Andrias Hojgaard, Michael Kosoy, Rebecca J. Eisen

**Affiliations:** ^1^ Bacterial Disease Branch Division of Vector‐Borne Diseases Centers for Disease Control and Prevention Fort Collins Colorado USA; ^2^ Department of Pathology Department of Microbiology & Immunology The University of Texas Medical Branch at Galveston Galveston Texas USA; ^3^ KB One Health LLC Fort Collins Colorado USA

**Keywords:** assay development, fleas, pentaplex real‐time PCR, *Yersinia pestis*, *Yersinia pseudotuberculosis*

## Abstract

Upon acquiring two unique plasmids (pMT1 and pPCP1) and genome rearrangement during the evolution from *Yersinia pseudotuberculosis*, the plague causative agent *Y*.* pestis* is closely related to *Y*.* pseudotuberculosis* genetically but became highly virulent. We developed a pentaplex real‐time PCR assay that not only detects both *Yersinia* species but also differentiates *Y*.* pestis* strains regarding their plasmid profiles. The five targets used were *Y*.* pestis*‐specific *ypo2088*, *caf1*, and *pst* located on the chromosome, plasmids pMT1 and pPCP1, respectively; *Y*.* pseudotuberculosis*‐specific chromosomal gene *opgG*; and 18S ribosomal RNA gene as an internal control for flea DNA. All targets showed 100% specificity and high sensitivity with limits of detection ranging from 1 fg to 100 fg, with *Y*.* pestis*‐specific *pst* as the most sensitive target. Using the assay, *Y*.* pestis* strains were differentiated 100% by their known plasmid profiles. Testing *Y*.* pestis* and *Y*.* pseudotuberculosis*‐spiked flea DNA showed there is no interference from flea DNA on the amplification of targeted genes. Finally, we applied the assay for testing 102 fleas collected from prairie dog burrows where prairie dog die‐off was reported months before flea collection. All flea DNA was amplified by 18S rRNA; no *Y*.* pseudotuberculosis* was detected; one flea was positive for all *Y*.* pestis*‐specific targets, confirming local *Y*.* pestis* transmission. Our results indicated the assay is sensitive and specific for the detection and differentiation of *Y*.* pestis* and *Y*.* pseudotuberculosis*. The assay can be used in field investigations for the rapid identification of the plague causative agent.

## Abstract 2

We developed a pentaplex real‐time PCR assay including *Yersinia pestis*‐specific genes ypo2088, caf1, and pst located on the chromosome, as well as on plasmids pMT1 and pPCP1, respectively. *Yersinia pseudotuberculosis*‐specific chromosomal gene opgG and the 18S ribosomal RNA gene served as an internal control for flea DNA. All targets showed 100% specificity and high sensitivity with limits of detection ranging from 1 to 100 femtograms. Using the assay, *Y. pestis* was detected in field‐collected fleas and strains were differentiated 100% by their known plasmid profiles.

## 1 INTRODUCTION

*Yersinia pestis*, a Tier 1 select agent, is the bacterial causative pathogen of a plague that infects rodents and can be transmitted to other mammals, including humans through flea biting (Perry & Fetherston, 1997; Stenseth et al., 2008). Plague causes serious illness or death if not treated promptly, especially if it develops into the pneumonic plague.

The plague has had a big impact on human history through three devastating pandemics (Perry & Fetherston, 1997) causing millions of deaths, as well as by locally endemic occurrence which results in hundreds to thousands of annual cases worldwide. Currently, the plague is endemic in Asia, Africa, and the Americas (Bertherat, 2016). Multiple recent outbreaks, including that in China, Democratic Republic of Congo, Madagascar, Peru, and Uganda (Abedi et al., 2018; Andrianaivoarimanana et al., 2019; Randremanana et al., 2019; Respicio‐Kingry et al., 2016; Shi et al., 2018) warn us not to ignore this severe infectious disease.

Along with *Y*.* pestis*, *Y*.* pseudotuberculosis*, and *Y*.* enterocolitica* are the other two related species within the genus *Yersinia* that can cause diseases in both animals and humans (Perry & Fetherston, 1997). Nevertheless, symptoms of disease caused by enteropathogens *Y*.* pseudotuberculosis* and *Y*.* enterocolitica* are vastly different from the plague. Belonging to the same genus, these organisms, especially *Y*.* pestis* and *Y*.* pseudotuberculosis*, are genetically closely related (Moore & Brubaker, 1975). Previous studies have indicated that *Y*.* pestis* has diverged recently from *Y*.* pseudotuberculosis* through events of genome reduction and gene gain; thus, the high genomic similarity were observed between the two species (Achtman et al., 1999; Brubaker, 2004; Chain et al., 2004; Hu et al., 1998; Moore & Brubaker, 1975; Parkhill et al., 2001). In the genetic structure, all three species possess a single circular chromosome and a common virulence plasmid termed pCD1, or pIB, or pYV in *Y*.* pestis*, *Y*.* pseudotuberculosis*, and *Y*.* enterocolitica*, respectively (Cornelis & Wolf‐Watz, 1997; Iriarte & Cornelis, 1996; Perry & Fetherston, 1997). The most prominent genetic difference between *Y*.* pestis* and the enteropathogenic *Yersinia* species is the presence of pPCP1 and pMT1, two newly acquired plasmids in most but not all strains of *Y*.* pestis* (Ben‐Gurion & Shafferman, 1981; Ferber & Brubaker, 1981; Filippov, Oleinikov, Motin, Protsenko, & Smirnov, 1995; Filippov, Solodovnikov, & Protsenko, 1990; Hu et al., 1998; Portnoy & Falkow, 1981). These plasmids contain different genes that play important roles regarding pathogenesis. The plasmid pCD1 contains the highly conserved type three secretion system (T3SS) injectosome and effector proteins Yops (Rajanna et al., 2010); pPCP1 contains *pla* gene and *pst* gene that encode plasminogen activator (PLA) protease and the pesticin protein (Bearden, Fetherston, & Perry, 1997); pMT1 contains *caf1* and *ymt*, encoding fraction 1 capsule antigen and murine toxin, respectively, as well as other putative virulence determinants (Hu et al., 1998).

Most *Y*.* pestis* strains isolated from humans, animals, or fleas contain all three typical plasmids. However, *Y*.* pestis* isolates lacking one or more plasmids may cause mild or even fatal disease, as previously been reported in South Africa and the United States (Beesley & Surgalla, 1970; Isaacson et al., 1973; Williams, Harrison, & Cavanaugh, 1975; Williams et al., 1978; Winter, Cherry, & Moody, 1960). Such variants have been reported infrequently. The natural occurrence of strains with atypical plasmid profiles might be greater than present data suggest.

The extreme pathogenicity of *Y*.* pestis* makes accurate and rapid detection of this bacterium a priority. Traditional methods of detecting *Y*.* pestis* include standard microbiological techniques (Norkina et al., 1994) and immunofluorescent staining (Feodorova & Devdariani, 2000). However, these methods take a long time and are relatively insensitive. In contrast, PCR‐based assays have the advantage of rapid detection with high sensitivity. Furthermore, PCR is often preferable with the select agent and biosafety concerns. PCR assays for *Y*.* pestis* detection are not uncommon. Several PCR assays have been developed (Campbell, Lowe, Walz, & Ezzell, 1993; Matero et al., 2009; Neubauer et al., 2000; Radnedge, Gamez‐Chin, McCready, Worsham, & Andersen, 2001; Riehm et al., 2011; Stewart, Satterfield, Cohen, O'Neill, & Robison, 2008; Tomaso et al., 2003; Woron et al., 2006). The pPCP1‐situated *pla* gene, known to be present in 150–200 copies per bacterium (Parkhill et al., 2001), has been widely used due to the very high sensitivity (Riehm et al., 2011; Stewart et al., 2008; Woron et al., 2006). Ideally, if we are dealing with typical *Y*.* pestis* strains that harbor all three plasmids, a *pla*‐based assay could be reasonable. However, a good portion (~25%) of *Y*.* pestis* strains existing in nature are deficient in one or more of the three plasmids (Filippov et al., 1990; Stewart et al., 2008) due to various host growth temperatures, as well as other unknown factors (Iqbal, Chambers, Goode, Valdes, & Brubaker, 2000). As such, a plasmid‐based assay may yield false‐negative results. On the other hand, a *pla* assay may cause false‐positive results because *pla* may also be present in other Enterobacteriaceae such as *Escherichia coli* and *Citrobacter koseri* (Armougom et al., 2016; Hänsch et al., 2015). Further, a homolog of *pla* has been reported in rodents of multiple species (Giles et al., 2016). In addition to plasmid‐based genes, chromosomal genes, such as *entF*3, were also applied for *Y*.* pestis* detection (Woron et al., 2006). The problem with using a chromosomal gene alone is it may involve non‐specific amplification due to the genetic relatedness of *Y*.* pestis* to the other *Yersinia* species. Amplification with a chromosomal gene may not indicate which species is present (Woron et al., 2006).

In the current study, we developed a pentaplex real‐time PCR assay that includes three *Y*.* pestis*‐specific targets, one *Y*.* pseudotuberculosis*‐specific target and one internal control for flea DNA since the assay is intended to test field‐collected fleas. The three *Y*.* pestis*‐specific targets in the assay are pPCP1‐situated *pst*, pMT1‐situated *caf1*, and chromosomal gene *ypo2088*. *ypo*2088 codes for a putative methyltransferase and is considered *Y*.* pestis*‐specific (Chain et al., 2004; Matero et al., 2009). The *Y*.* pseudotuberculosis*‐specific gene used in the assay is chromosomal gene *opgG*. *Yersinia pestis* lost this gene during its speciation from *Y*.* pseudotuberculosis*; hence, *opgG* can differentiate *Y*.* pestis* strains and *Y*.* pseudotuberculosis* strains (Quintard et al., 2015). For the internal control of flea DNA, the very conservative 18S ribosomal gene was used for a wide application for any flea species in general. The objective of this study was to allow accurate and rapid identification of all *Y*.* pestis* strains from field‐collected fleas; to discriminate *Y*.* pestis* from *Y*.* pseudotuberculosis*; and to differentiate *Y*.* pestis* strains in regard to their plasmid profiles.

## 2 EXPERIMENTAL PROCEDURES

### 2.1 Gene selection, primer, and probe design

Five genes were selected for developing the pentaplex real‐time assay: *Yersinia pst*, *caf1*, *ypo2088*, and *opgG*, and flea 18S rRNA. Sequences of primer and probe used in the assay were designed for the current study except for those for *caf1* which were adapted from assays published (Liu et al., 2016) (Table 1). The TaqMan primers/probes design was conducted with the PrimerQuest program, Integrated DNA Technologies, Inc., Coralville, Iowa, https://www.idtdna.com/SciTools. Also, the *pla* gene of *Y*.* pestis* was tested using previously published primers and probe (Riehm et al., 2011) for comparison and validation purposes.

**Table 1:** Description of the genes selected for the pentaplex real‐time PCR and the validation PCR, and the primers/probes sequences

Gene/location	Detection	Primer/probe sequences (5′‐3′)	Product	Reference
*pst*/pPCP1	*Y*.* pestis* strains with pPCP1 plasmid presence	Forward: GCGAAGCAAACAGGATTTATTG	116 bp	This study
		Reverse: GAGGTGCTGTTCTCACTTTATC		
		Probe: FAM‐AGCCTCCTTCCCTCGAAGCATATAATACCC‐BHQ−1		
*caf1*/pMT1	*Y*.* pestis* strains with pMT1 plasmid presence	Forward: CCACTGCAACGGCAACTCTT	71 bp	Liu et al. (2016)
		Reverse: TGTAATTGGAGCGCCTTCCT		
		Probe: QUAS705‐TTGAACCAGCCCGCATCACTCTTACA‐BHQ3		
*ypo2088*/chromosome	All *Y*.* pestis* strains	Forward: TCGGCAACAGCTCAACACCT	107 bp	This study
		Reverse: ATGCATTGGACGGCATCACG		
		Probe: CALRD610‐CGCCCTCGAATCGCTGGCCAACTGC‐BHQ2		
*opgG*/chromosome	*Y*.* pseudotuberculosis* strains	Forward: ACGTGGGCGTGAATTCTCTCAA	126 bp	This study
		Reverse: GCCGTTGGGATCTCCACCAA		
		Probe: QUAS670‐CCTGCGCCCAAGCGCGTGGG‐BHQ2		
18S rRNA	Internal control for flea DNA	CAGATACCGCCCTAGTTC TAA	135 bp	This study
		Reverse: GTTTCAGCTTTGCAACCATAC		
		Probe: HEX‐TCATCGGAGGAACTTCGGCGGATC‐BHQ−1		
*pla*/pPCP1	*Y*.* pestis* strains with pPCP1 plasmid presence	Forward: Forward: GTAATAGGTTATAACCAGCGCTT	232 bp	Rajanna et al. (2010) and Tomaso et al. (2003)
		Reverse: AGACTTTGGCATTAGGTGTG		
		Probe: HEX‐ATGCCATATATTGGACTTGCAGGCCAGT‐BHQ1		

### 2.2 Template DNA

All *Y*.* pestis* and *Y*.* pseudotuberculosis* DNA templates used in the study were obtained from BEI Resources or the University of Texas Medical Branch (UTMB) unless otherwise stated (Table 2). *Yersinia pestis*: CO96‐3188 DNA was obtained from CDC Bacterial Diseases Branch. CO96‐3188 is a fully virulent (Pgm+, pMT1+, pCD1+, and pPCP1+) North American biovar Orientalis strain (Eisen et al., 2006; Engelthaler, Hinnebusch, Rittner, & Gage, 2000). For the other 16 *Y*.* pestis* strains, both pMT1 and pPCP1 were present in 11 strains; both pMT1 and pPCP1 were absent in one strain; pPCP1was absent in four more strains (Table 2). *Yersinia pseudotuberculosis* was represented by strain B15 and four other strains (Table 2). Two *Y*.* enterocolitica* strains were included for specificity checking (Table 2). Also, we included 12 non‐*Yersinia* species representing five *Bartonella* spp. (*B*.* bovis*, *B*.* doshiae*, *B*.* elizabethae*, *B*.* henselae*, and *B*.* rochalimae*), *Rickettsia rickettsii*, *Anaplasma phagocytophilum*, three *Borrelia* spp. (*B*.* burgdorferi*, *B*.* miyamotoi*, *B*.* mayonii*), *Trypanosoma cruzi*, and *Leptospira interrogans* for specificity testing (Table 3). The reason we included these species is that the transmission route of these bacteria is similar to the *Yersinia* species, either by fleas or other blood‐feeding vectors (Bai et al., 2017; Brook et al., 2015; Livengood, Hutchinson, Thirumalapura, & Tewari, 2020; Pawelczyk, Asman, & Solarz, 2019) or by consumption of contaminated water (Casanovas‐Massana, Pedra, Wunder, Begon, & Ko, 2018). All DNA from these non‐*Yesinia* species was obtained from CDC Bacterial Diseases Branch. DNA extracted from *Xenopsylla cheopis* fleas raised at the CDC colony were used as internal controls in the experiment. Finally, DNA extracted from alcohol‐preserved field‐collected fleas were tested to check whether 18S rRNA amplifies DNA of flea species other than *X*.* cheopis*, and if interference or inhibition would occur while applying the assay to field samples. These fleas were collected from guinea pigs (*Cavia porcellus*) in Peru and represent three species, including *Pulex* sp. (*n* = 6), *Ctenocephalides felis* (*n* = 5), and *Tiamastus cavicola* (*n* = 5). A previous study showed they were negative for *Bartonella* species (María et al., 2019).

**Table 2:** Plasmid presence/absence in *Yersinia pestis* strains and PCR results in all strains of *Y*.* pestis*,* Y*.* pesudotuberculosis*, and *Y*.* enterocolitica* used to evaluate assay performance

ID	Strain	Source	Plasmid presence		PCR			
			pMT1	pPCP1	*ypo2088*	*caf1*	*pst*	*opgG*
*Y. pestis*								
NR‐4709	KIM D23	BEI	Yes	No	pos	pos	neg	neg
NR‐4713	A12 D6	BEI	Yes	No	pos	pos	neg	neg
NR‐4706	KIM D2	BEI	Yes	Yes	pos	pos	pos	neg
NR‐4717	Yokohama D11	BEI	Yes	Yes	pos	pos	pos	neg
NR‐4719	Kimberley D13	BEI	Yes	Yes	pos	pos	pos	neg
NR‐4727	K25 D80	BEI	Yes	Yes	pos	pos	pos	neg
NR‐4705	KIM D19	BEI	Yes	Yes	pos	pos	pos	neg
NR‐2645	KIM10+	BEI	Yes	No	pos	pos	neg	neg
NR‐4708	KIM D22	BEI	Yes	No	pos	pos	neg	neg
NR‐4714	Kuma D7	BEI	Yes	Yes	pos	pos	pos	neg
NR‐4716	yokohama D10	BEI	Yes	Yes	pos	pos	pos	neg
NR‐4718	Kimberley D12	BEI	Yes	Yes	pos	pos	pos	neg
NR‐4721	K25 D72	BEI	Yes	Yes	pos	pos	pos	neg
NR‐2718	PB6	BEI	No	No	pos	neg	neg	neg
NR‐2719	Harbin 35	BEI	Yes	Yes	pos	pos	pos	neg
NR‐2720	Nepal 516	BEI	Yes	Yes	pos	pos	pos	neg
*Y. pseudotuberculosis*					neg	neg	neg	pos
NR‐4653	YPIII(p+)	BEI			neg	neg	neg	pos
NR‐4651	IP2775	BEI			neg	neg	neg	pos
4284	4284	UTMB			neg	neg	neg	pos
6904	6904	UTMB			neg	neg	neg	pos
*Y. enterocolitica*								
NR‐3064	Billups‐1803‐68	BEI			neg	neg	neg	neg
NR‐3065	WA	BEI			neg	neg	neg	neg

**Table 3:** PCR results on non‐*Yersinia* bacterial species for specificity testing of the pentaplex assay

Bacterial species	*YPO2088*	*caf1*	*pst*	*opgG*	18S rRNA
*Anaplasma phagocytophilum*	neg	neg	neg	neg	neg
*Bartonella bovis*	neg	neg	neg	neg	neg
*Bartonella doshiae*	neg	neg	neg	neg	neg
*Bartonella elizabethae*	neg	neg	neg	neg	neg
*Bartonella henselae*	neg	neg	neg	neg	neg
*Bartonella rochalimae*	neg	neg	neg	neg	neg
*Borrelia bergdorferi*	neg	neg	neg	neg	neg
*Borrelia mayonii*	neg	neg	neg	neg	neg
*Borrelia miyamotoi*	neg	neg	neg	neg	neg
*Leptospira interrogans*	neg	neg	neg	neg	neg
*Rickettsia rickettsii*	neg	neg	neg	neg	neg
*Trypanosoma cruzi*	neg	neg	neg	neg	neg

### 2.3 Optimization of primer concentration

DNA of *Y*.* pestis* wild‐type strain CO96‐3188, *Y*.* pseudotuberculosis* strain B15 and *Xenopsylla cheopis* fleas raised at the CDC colony were used for the optimization of the primer concentration and identification of the limit of detection (LOD) of each target. Three DNA concentrations (100 pg/µl, 10 pg/µl, 1 pg/µl) of each template were used to test the targets independently with the corresponding primers at different concentrations, for example, DNA of *Y*.* pestis* strain CO96‐3188 for *pst*, *caf1*, and *ypo2088*; DNA of *Y*.* pseudotuberculosis* strain B15 for *opgG*; and DNA of *Xenopsylla cheopis* flea for 18S rRNA. The optimal primer concentrations were tested using a primer concentration matrix with final concentrations of 200 nM–1000 nM. PCR (25 µl) contained 12.5 µl of 2× PerfeCta MultiPlex qPCR SuperMix (Quanta Biosciences, Gaithersburg, MD), primers (forward and reverse), probes at a standard concentration of 200 nM, and 5 µl of DNA template. Real‐time PCR was performed on a CFX96 Real‐Time System (Bio‐Rad, Hercules, CA) with the following conditions: 95°C for 2 min followed by 45 cycles of 95°C for 15 s and 64°C for 60 s. Distilled water was always included as a negative control. A primer concentration that generates a sigmoidal shape of the amplification curve with a relatively lower *Ct* value compared to other primer concentration is considered an optimized concentration.

### 2.4 Sensitivity testing and specificity assessment

Sensitivity was estimated using the limit of detection (LOD). The LOD for each target was determined and verified by comparing the primer/probe in a single PCR and in a pentaplex PCR simultaneously using the optimized primer concentration by testing six replicates of each DNA template with the following dilutions: 2 pg, 1 pg, 0.5 pg, 0.2 pg, 0.1 pg, 50 fg, 20 fg, 10 fg, 5 fg, 2 fg, 1 fg, 0.5 fg, 0.2 fg, 0.1 fg, 0.05 fg, and 0.02 fg. The lowest dilution that was amplified in all six replicates in both single PCR and pentaplex PCR, with a threshold *Ct* value <40 in any of the reactions was considered the LOD. To validate the result, an identical set of real‐time PCR was performed using the widely used *pla* gene (Riehm et al., 2011).

The specificity of each primer/probe set was assessed by testing high concentration (500 pg) of nucleic acid from *Y*.* pestis*, *Y*.* pseudotuberculosis*, *Y*.* enterocolitica* strains, and other 12 non‐*Yersinia* microorganisms (Table 3) by running a pentaplex real‐time PCR using the optimized primer concentrations.

### 2.5 Evaluation of the pentaplex assay

To evaluate whether the pentaplex assay is applicable for real sample testing, sixteen fleas collected from guinea pigs in Peru and were preserved in alcohol before DNA extraction were first tested for the presence of flea DNA and any other DNA using the pentaplex PCR assay; then, the flea DNA was spiked with DNA of *Y*.* pestis* strain CO96‐3788 or *Y*.* pseudotuberculosis* strain B15. For comparison purposes, final DNA concentrations of *Y*.* pestis* strain or *Y*.* pseudotuberculosis* matched those used for sensitivity testing. Pentaplex PCR was performed to test the *Y*.* pestis*‐ and *Y*.* pseudotuberculosis*‐spiked flea DNA separately to check whether the flea DNA and alcohol would interfere or inhibit the amplification. Six replicates were tested for each concentration of each spiked DNA.

### 2.6 Differentiation of *Yersinia* species and elucidation of *Y*.* pestis* strains with different plasmid profiles

Genomic DNA of *Y*.* pestis* strains with different plasmid profiles, *Y*.* pseudotuberculosis* strains, and *Y*.* enterocolitica* strains were tested with all four sets of *Yersinia* primer/probe (*pst*, *caf1*, *ypo2088*, and *opgG*) with a correspondent optimized concentration of each primer in a multiplex PCR setting.

### 2.7 Application of the assay for testing field‐collected fleas

To evaluate the utility of the pentaplex real‐time PCR assay, we tested fleas (*n* = 102) collected from burrows in a prairie dog colony in Converse County, Wyoming in July 2019. A prairie dog die‐off was noticed in the area in February 2019 and plague activity was suspected. Collected fleas belonged to three species: *Oropsylla hirsuta* (*n* = 87), *O*.* tuberculata* (*n* = 14), and *Thrassiis fotus* (*n* = 1). The fleas were stored in 70% ethanol and kept at 4°C before testing. DNA extraction was prepared from individual fleas using a Kingfisher platform (Life Technologies, Inc., Grand Island, NY) following the instructions provided by the manufacturer.

## 3 RESULTS

### 3.1 Optimization of primer concentration

**Figure 1:**
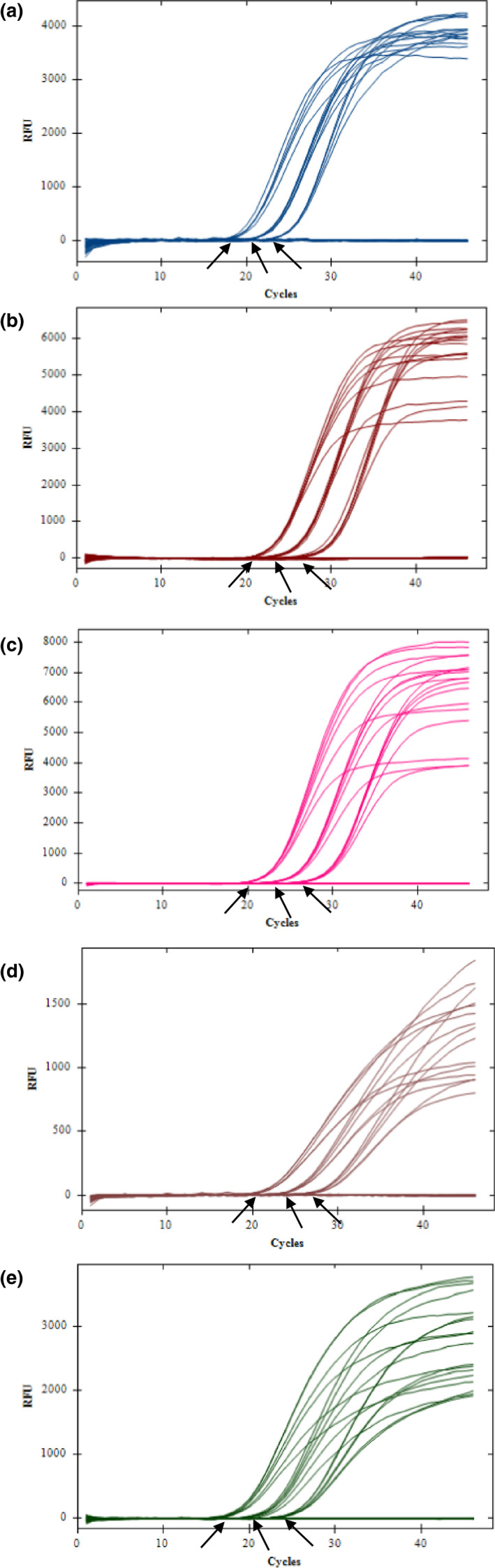
Amplification of DNA (100 pg/µl, 10 pg/µl, 1 pg/µl) with different primer concentrations ranging from 200 nM to 1000 nM. The amplification was grouped tightly by the concentration of DNA. The higher the DNA concentration, the earlier the amplification started (lower *Ct* value). Primer concentrations had no significant differences in the amplification efficiencies with a very small change of *Ct* value. The three arrows indicated the DNA concentration for each group was 100 pg/µl, 10 pg/µl, and 1 pg/µl from left to right. A. *pst*; B. *caf1*; C. *ypo2088*; D. *opgG*; E. 18S rRNA

The amplification was grouped tightly by the concentration of DNA for each target (Figure 1). The higher the DNA concentration, the earlier the amplification started (lower *Ct* value). Primer concentrations had no significant differences in the amplification efficiencies with the change of *Ct* value <1. For example, for *pst* gene, the *Ct* value ranged 22.14–22.82 with primer concentrations of 200 nM–1000 nM when the DNA concentration was 100 pg. Based on our criteria (generating a sigmoidal shape of the amplification curve with lower *Ct* value), the optimized primer concentration is 200 nM for all *Y*.* pestis*‐associated targets (*pst*, *caf1*, and *ypo2088*) primers, 600 nM for *opgG* primers, and 500 nM for 18S rRNA primers (Table 4).

**Table 4:** Optimization of primer/probe concentration and identification of limit of detection (LOD) of each gene used in the pentaplex assay

Gene	Primer concentration (nM)	Probe concentration (nM)	LOD (fg)
*pst*	200 nM	200 nM	1
*caf1*	200 nM	200 nM	50
*ypo2088*	200 nM	200 nM	100
*opgG*	600 nM	200 nM	5
18S rRNA	500 nM	200 nM	1

### 3.2 Sensitivity and specificity

**Figure 2:**
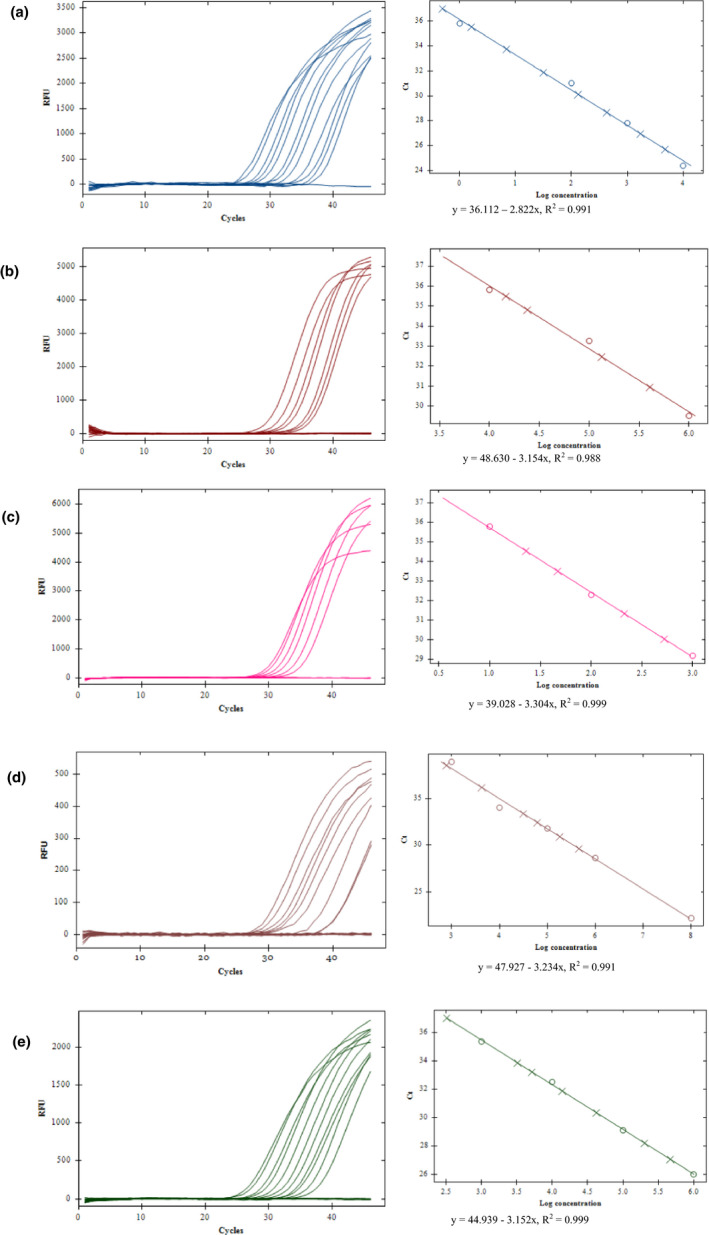
Sensitivity testing. Amplification (left panel) and standard curve (right panel) of each target with DNA dilutions 2 pg, 1 pg, 0.5 pg, 0.2 pg, 0.1 pg, 50 fg, 20 fg, 10 fg, 5 fg, 2 fg, 1 fg, 0.5 fg, 0.2 fg, 0.1 fg, 0.05 fg, and 0.02 fg. *Ct* values were correlated to the DNA concentration for each target. A. *pst*; B. *caf1*; C. *ypo2088*; D. *opgG*; E. 18S rRNA

All targets showed extremely high sensitivity (Figure 2). Among the three *Y*.* pestis*‐specific targets, *pst* was the most sensitive with a LOD of 1 fg per reaction, followed by *caf1* (LOD 50 fg) and *ypo2088* (LOD 100 fg) (Table 4). The *Y*.* pseudotuberculosis*‐specific *opgG* also showed very high sensitivity, with a LOD of 5 fg per reaction; the internal control target 18S rRNA for flea DNA had a LOD of 1 fg. Standard curves showed the *Ct* values were correlated to the DNA concentration for each target (Figure 2).

To validate the above testing results, a PCR was performed using the widely used *pla* gene. The PCR detected 1 fg *Y*.* pestis* DNA as the LOD, which is identical to that of *pst*, suggesting that *pst* gene and *pla* gene are equivalent in terms of the sensitivity.

All primer/probe sets showed 100% specificity to the targets accordingly. DNA of *Y*.* pestis* strain CO96‐3188 was positive to *pst*, *caf1*, and *ypo*2088 but negative to *opgG* and 18S rRNA; DNA of *Y*.* pseudotuberculosis* strain B15 was positive to *opgG* but negative to all other genes; DNA of *X*.* cheopis* fleas was positive to 18S rRNA but negative to all other genes; no amplification to any of the genes was observed in *Y*.* enterocolitica* (Table 2) and any of the other 12 species of non‐*Yersinia* microorganisms (Table 3).

### 3.3 Differentiation between *Y*.* pestis* strains and other *Yersinia* species

Testing of all *Yersinia* DNA (16 *Y*.* pestis*, 4 *Y*.* pseudotuberculosis*, and 2 *Y*.* enterocolitica*) with the four primers/probe sets of *Yersinia* targets demonstrated that the two *Y*.* enterocolitica* were negative to all targets; the four *Y*.* pseudotuberculosis* were positive to *Y*.* pseudotuberculosis*‐specific *opgG* but to none of the *Y*.* pestis*‐specific genes; the 16 *Y*.* pestis* DNA samples were positive to different *Y*.* pestis*‐specific targets, which was associated with their plasmid profile (BEI resource): All strains were positive to the chromosomal gene *ypo2088*; the strain with pMT1 and pPCP1 absent was negative to both *caf1* and *pst*; four strains with pPCP1 absence were negative to *pst*. None of the *Y*.* pestis* strains were positive to *opgG* (Table 2).

### 3.4 Pentaplex assay evaluation

Testing of 16 DNA of fleas collected from guinea pigs in Peru showed all fleas were amplified with 18S rRNA, with *Ct* values ranging 17.62–31.08 for the *Pulex* fleas; 20.54–34.33 for the *Ctenocephalides felis* fleas; and 21.13–31.05 for the *Tiamastus cavicola* fleas, showing that 18S rRNA worked equally for different flea species. All fleas were negative to *ypo2088*, *pst*, *caf1*, and *opgG*.

Testing of *Y*.* pestis* DNA‐spiked flea DNA showed that *ypo2088*, *pst*, *caf1*, and 18S rRNA were amplified, with LOD 1 fg, 50 fg, 100 fg for *ypo2088*, *pst*, *caf1*, respectively, all of which were identical to the LOD observed in the sensitive testing. All DNA in this group was negative to *opgG*.

Testing of *Y*.* pseudotuberculosis* DNA‐spiked flea DNA showed that *opgG* and 18S rRNA were amplified with LOD 5 fg for *opg*. The LOD was identical to that observed in the sensitive testing. All DNA in this group was negative to *ypo2088*, *pst*, and *caf1*.

### 3.5 Detection of *Yersinia pestis* in field‐collected fleas

**Figure 3:**
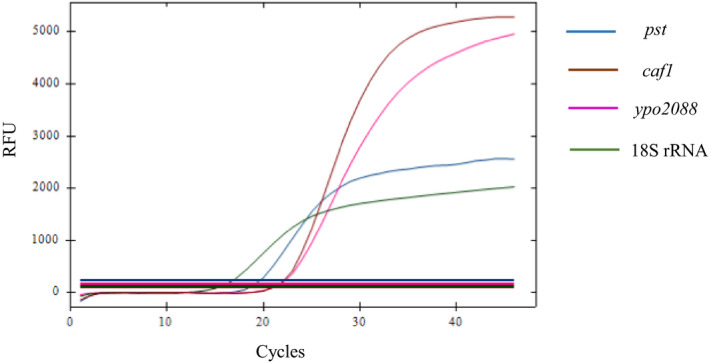
By performing the pentaplex real‐time PCR, one *Oropsylla tuberculata* flea collected from prairie dog burrows after a prairie dog die‐off in Wyoming in 2019 was positive to *pst*, *caf1*, *ypo2088*, and 18S rRNA, with *Ct* value 19.65, 21.08, 21.74, and 15.06, respectively, suggesting a local *Y*.* pestis* transmission

By performing the pentaplex real‐time PCR, all 102 fleas collected from prairie dog burrows several months after a prairie dog die‐off was reported in Converse County, Wyoming in July 2019 were positive to 18S rRNA, with *Ct* value ranging 13.93–27.91. Among these, one *Oropsylla tuberculata* flea (*Ct* value 15.06 for 18S rRNA) was positive to all three *Y*.* pestis* genes, with *Ct* value 19.65, 21.08, and 21.74 for *pst*, *caf1*, and *ypo2088*, respectively (Figure 3). The other 101 fleas were negative to any of the three *Y*.* pestis* genes. No *Y*.* pseudotuberculosis* was detected in any fleas all of which was negative to *opgG*.

## 4 DISCUSSION

It is of supreme importance using analytical assays for *Y*.* pestis* which can show the presence of various targets located on the chromosome and plasmids. A pentaplex real‐time PCR assay developed in this study included three *Y*.* pestis*‐specific genes (*pst*, *caf1*, and *ypo2088*), one *Y*.* pseudotuberculosis* gene (*opgG*), and 18S rRNA as an internal control of flea DNA if the assay is to be applied to test fleas. Multiplex PCR assays for *Y*.* pestis* are not uncommon (Stewart et al., 2008; Tomaso et al., 2003; Woron et al., 2006) but, to our knowledge, this is the first to integrate five biologically meaningful targets.

Many assays have used the *pla* gene that is located on the pPCP1 plasmid for the detection of *Y*.* pestis* due to its high sensitivity with the presence of high copy numbers (150–200 copies) (Parkhill et al., 2001). Finding a homolog of *pla* in other bacteria or rodents (Armougom et al., 2016; Giles et al., 2016; Hänsch et al., 2015) has raised a concern of false identification, particularly when considering the frequency of flea‐feeding on rodent hosts. Instead of using *pla*, we used *pst* which is also located on pPCP1. Our results demonstrated *pst* is equally as sensitive as the *pla* gene with the same LOD (1 fg per reaction). Such results suggest that *pst* is a plausible diagnostic marker to replace *pla*. Like *pla*, the pMT1‐located *caf1* is another commonly used target for *Y*.* pestis* detection in multiple studies (Norkina et al., 1994; Stewart et al., 2008; Tomaso et al., 2003; Woron et al., 2006). Similar to other reports, our results also showed that *caf1* is highly sensitive, but somewhat less sensitive compared to *pst*. It is known that *caf1* is present in only about two copies per bacterium (Parkhill et al., 2001), explaining the differences in sensitivity between the two genes.

We did not use any pCD1‐located genes in this study. This is because pCD1 is possessed by all currently recognized pathogenic *Yersinia* species. Detection based on pCD1‐located genes does not necessarily indicate the species of the presenting organism as previous studies have shown detection of *Y*.* pestis*, *Y*.* enterocolitica*, and *Y*.* pseudotuberculosis* using pCD1‐located *lcrV* or other targets (Stewart et al., 2008). Further, using a pCD1‐located gene may not be as efficient compared to pPCP1‐ and pMT1‐located genes because the pCD1 plasmid is absent in many more *Y*.* pestis* strains compared to pPCP1 and pMT1 plasmids in nature due to the intrinsic variability of pCD1 plasmids in *Y*.* pestis* (Stewart et al., 2008).

Unlike plasmids, chromosomal genes are not likely to be lost during laboratory cultivation or in nature; thus, chromosomal genes are more reliable targets than plasmid genes. Nevertheless, specific identification can be difficult if substantial genomic similarities are shared among multiple species. In the past, researchers have used the *inv* gene, the *entF3* gene, the *wzz* gene, and the 16S rRNA gene in the identification of *Y*.* pestis* (Matero et al., 2009; Neubauer et al., 2000; Tomaso et al., 2003; Woron et al., 2006). However, non‐specific amplification has been observed with these genes. All of them not only amplify *Y*.* pestis*, but also *Y*.* pseudotuberculosis*. In our assay, we used *ypo2088* and *opgG*, two chromosomal genes that are designed for *Y*.* pestis* and *Y*.* pseudotuberculosis*, respectively. Our results demonstrated that all *Y*.* pestis* strains were detected by *ypo2088* and *Y*.* pseudotuberculosis* strains by *opgG*. Both *ypo2088* and *opgG* showed high sensitivity (LOD 100 fg and 5 fg respectively) with 100% specificity to their targeted *Yersinia* species. Because *opgG* has been lost in *Y*.* pestis*, this makes it ideal for the differentiation of *Y*.* pestis* and *Y*.* pseudotuberculosis*. With a combined analysis of *Y*.* pestis*‐specific and *Y*.* pseudotuberculosis*‐specific assays, any detection will be elucidated by the exclusion of one from the other. No amplification was observed with *Y*.* enterocolitica* to either gene. Also, several non‐*Yersinia* microorganisms were tested using the assay for specificity assessment with no amplification observed in any of them. Our results demonstrated all primer/probe used in the assay was 100% specific.

Using the developed assay, we tested *Y*.* pestis* strains with known plasmid profiles (BEI Resource). All strains were successfully identified by *pst* and *caf1* in accordance with the presence of the pPCP1 plasmid and pMT1 plasmid with 100% specificity.

Laboratory‐based PCR assays may struggle with different problems in reality, such as low concentration, and inhibition. To observe if that would be a case for our pentaplex assay, we spiked *Y*.* pestis* DNA and *Y*.* pseudotuberculosis* DNA into DNA of fleas that were collected from guinea pigs in Peru and preserved in alcohol before DNA extraction. The results showed the LOD of each primer/probe set on the *Y*.* pestis*‐ and *Y*.* pseudotuberculosis*‐spiked flea DNA was the same as that observed in the sensitive testing, suggesting no interference or inhibition from fleas or alcohol on the amplifications.

We applied the assay to test 102 fleas that were collected from prairie dog burrows where prairie dog die‐offs were reported 5 months before our field investigation. All fleas were successfully amplified with the 18S rRNA. One out of the 102 fleas was positive for *Y*.* pestis* by all three *Y*.* pestis*‐specific genes (*pst*, *caf1*, and *ypo2088*). The result confirmed the local transmission of *Y*.* pestis* in the prairie dog colony. Since the flea collection did not occur until 5 months afterward the prairie dog die‐off, the low number of positive samples may have suggested plague activity had diminished gradually after the epizootic peak. Flea numbers were noticeably lower compared to an earlier investigation conducted in the area (around 3 months after the die‐off). This indicated that most fleas may have died of starvation. The extremely low number of positive fleas was reasonably expected (Tripp, Gage, Montenieri, & Antolin, 2009). Not surprisingly, no *Y*.* pseudotuberculosis* was detected in any of these fleas.

In conclusion, the pentaplex real‐time PCR assay developed in this work is highly sensitive and 100% specific in the detection and differentiation of *Y*.* pestis* and *Y*.* pseudotuberculosis*. Further, the assay allows one for the elucidation of the presence/absence of *Y*.* pestis* pPCP1 plasmid and pMT1 plasmid in a particular strain, which can be applied to testing fleas and other field‐collected materials when the plague is suspected.

## CONFLICT OF INTEREST

The conclusions, findings, and opinions expressed by authors contributing to this journal do not necessarily reflect the official position of the U.S. Department of Health and Human Services, the Public Health Service, the Centers for Disease Control and Prevention, or the authors' affiliated institutions.

## AUTHOR CONTRIBUTIONS

**Ying Bai:** Conceptualization (lead); data curation (lead); formal analysis (lead); investigation (lead); methodology (lead); project administration (equal); supervision (lead); validation (lead); visualization (lead); writing‐original draft (lead); writing‐review and editing (lead). **Vladimir Motin:** Conceptualization (lead); methodology (lead); writing‐review and editing (equal). **Russell E. Enscore:** Investigation (lead); writing‐review and editing (supporting). **Lynn Osikowicz:** Investigation (equal). **Maria Rosales Rizzo:** Investigation (equal). **Andrias Hojgaard:** Methodology (equal). **Michael Kosoy:** Conceptualization (equal); writing‐review and editing (equal). **Rebecca J. Eisen:** Writing‐review and editing (equal).

## ETHICS STATEMENT

None required.

## Data Availability

All data generated or analyzed during this study are included in this published article.

## References

[mbo31105-bib-0001] Abedi, A. A. , Shako, J.‐C. , Gaudart, J. , Sudre, B. , Ilunga, B. K. , Shamamba, S. K. B. , Diatta, G. , … Piarroux, M. (2018). Ecologic features of plague outbreak areas, Democratic Republic of the Congo, 2004–2014. Emerging Infectious Diseases, 24, 210–220. 29350136 10.3201/eid2402.160122 PMC5782875

[mbo31105-bib-0002] Achtman, M. , Zurth, K. , Morelli, G. , Torrea, G. , Guiyoule, A. , & Carniel, E. (1999). *Yersinia pestis*, the cause of plague, is a recently emerged clone of *Yersinia pseudotuberculosis* . Proceedings of the National Academy of Sciences of the United States of America, 96, 14043–14048. 10570195 10.1073/pnas.96.24.14043 PMC24187

[mbo31105-bib-0003] Andrianaivoarimanana, V. , Piola, P. , Wagner, D. M. , Rakotomanana, F. , Maheriniaina, V. , Andrianalimanana, S. , … Rajerison, M. (2019). Trends of human plague, Madagascar, 1998–2016. Emerging Infectious Diseases, 25, 220–228. 30666930 10.3201/eid2502.171974 PMC6346457

[mbo31105-bib-0004] Armougom, F. , Bitam, I. , Croce, O. , Merhej, V. , Barassi, L. , Nguyen, T. T. , … Raoult, D. (2016). Genomic insights into a new *Citrobacter koseri* strain revealed gene exchanges with the virulence‐associated *Yersinia pestis* pPCP1 plasmid. Frontiers in Microbiology, 7, 340 10.3389/fmicb.2016.00340 27014253 PMC4793686

[mbo31105-bib-0005] Bai, Y. , Osikowicz, L. M. , Kosoy, M. Y. , Eisen, R. J. , Atiku, L. A. , Mpanga, J. T. , Gage, K. (2017). Comparison of zoonotic bacterial agents in fleas collected from small mammals or host‐seeking fleas from a Ugandan region where plague is endemic. mSphere, 2, e00402‐17 10.1128/mSphere.00402-17 29276773 PMC5737051

[mbo31105-bib-0006] Bearden, S. W. , Fetherston, J. D. , & Perry, R. (1997). Genetic organization of the yersiniabactin biosynthetic region and construction of avirulent mutants in *Yersinia pestis* . Infection and Immunity, 65, 1659–1668. 9125544 10.1128/iai.65.5.1659-1668.1997 PMC175193

[mbo31105-bib-0007] Beesley, E. D. , & Surgalla, M. J. (1970). Pesticinogeny: A characteristic useful for presumptive identification and isolation of Pasteurella pestis. Applied Microbiology, 19, 915–918. 4989537 10.1128/am.19.6.915-918.1970 PMC376823

[mbo31105-bib-0008] Ben‐Gurion, R. , & Shafferman, A. (1981). Essential virulence determinants of different *Yersinia* species are carried on a common plasmid. Plasmid, 5, 183–187. 7243971 10.1016/0147-619x(81)90019-6

[mbo31105-bib-0009] Bertherat, E. (2016). Plague around the world, 2010–2015. Weekly Epidemiological Record, 91, 89–104.26922822

[mbo31105-bib-0010] Brook, C. E. , Bai, Y. , Dobson, A. P. , Osikowicz, L. M. , Ranaivoson, H. C. , Zhu, Q. , … Dittmar, K. (2015). Bartonella spp. in fruit bats and blood‐feeding Ectoparasites in Madagascar. PLOS Neglected Tropical Diseases, 9, e0003532 10.1371/journal.pntd.0003532. 25706653 PMC4337899

[mbo31105-bib-0011] Brubaker, R. R. (2004). The recent emergence of plague: A process of felonious evolution. Microbial Ecology, 47, 293–299. 15037962 10.1007/s00248-003-1022-y

[mbo31105-bib-0012] Campbell, J. , Lowe, J. , Walz, S. , & Ezzell, J. (1993). Rapid and specific identification of *Yersinia pestis* by using a nested polymerase chain reaction procedure. Journal of Clinical Microbiology, 31, 758–759. 8458980 10.1128/jcm.31.3.758-759.1993 PMC262866

[mbo31105-bib-0013] Casanovas‐Massana, A. , Pedra, G. G. , Wunder, E. A., Jr. , Diggle, P. J. , Begon, M. , & Ko, A. I. (2018). Quantification of leptospira interrogans survival in soil and water microcosms. Applied and Environment Microbiology, 84, e00507‐18 10.1128/AEM.00507-18 PMC6007094 29703737

[mbo31105-bib-0014] Chain, P. S. , Carniel, E. , Larimer, F. W. , Lamerdin, J. , Stoutland, P. O. , Regala, W. M. , … Garcia, E. (2004). Insights into the evolution of *Yersinia pestis* through whole‐genome comparison with Yersinia pseudotuberculosis. Proceedings of the National Academy of Sciences of the United States of America, 101, 13826–13831. 15358858 10.1073/pnas.0404012101 PMC518763

[mbo31105-bib-0015] Cornelis, G. R. , & Wolf‐Watz, H. (1997). The *Yersinia* Yop virulon: a bacterial system for subverting eukaryotic cells. Molecular Microbiology, 23, 861–867. 9076724 10.1046/j.1365-2958.1997.2731623.x

[mbo31105-bib-0016] Eisen, R. J. , Bearden, S. W. , Wilder, A. P. , Montenieri, J. A. , Antolin, M. F. , & Gage, K. L. (2006). Early‐phase transmission of Yersinia pestis by unblocked fleas as a mechanism explaining rapidly spreading plague epizootics. Proceedings of the National Academy of Sciences of the United States of America, 103, 15380–15385. 17032761 10.1073/pnas.0606831103 PMC1592641

[mbo31105-bib-0017] Engelthaler, D. M. , Hinnebusch, B. J. , Rittner, C. M. , & Gage, K. L. (2000). Quantitative competitive PCR as a technique for exploring flea‐Yersina pestis dynamics. American Journal of Tropical Medicine and Hygiene, 62, 552–560. 11289663 10.4269/ajtmh.2000.62.552

[mbo31105-bib-0018] Feodorova, V. A. , & Devdariani, Z. L. (2000). Development, characterisation and diagnostic application of monoclonal antibodies against *Yersinia pestis* fibrinolysin and coagulase. Journal of Medical Microbiology, 49, 261–269. 10707946 10.1099/0022-1317-49-3-261

[mbo31105-bib-0019] Ferber, D. M. , & Brubaker, R. R. (1981). Plasmids in *Yersinia pestis* . Infection and Immunity, 31, 839–841. 7216478 10.1128/iai.31.2.839-841.1981 PMC351389

[mbo31105-bib-0020] Filippov, A. A. , Oleinikov, P. N. , Motin, V. L. , Protsenko, O. A. , & Smirnov, G. B. (1995). Sequencing of two *Yersinia pestis* IS elements, IS285 and IS100. Contributions to Microbiology and Immunology, 13, 306–309. 8833859

[mbo31105-bib-0021] Filippov, A. A. , Solodovnikov, N. S. , & Protsenko, O. A. (1990). Plasmid content in *Yersinia pestis* strains of different origin. FEMS Microbiology Letters, 67, 45–48. 10.1016/0378-1097(90)90165-m 2328909

[mbo31105-bib-0022] Giles, T. A. , Greenwood, A. D. , Tsangaras, K. , Giles, T. C. , Barrow, P. A. , Hannant, D. , … Yon, L. (2016). Detection of a *Yersinia pestis* gene homologue in rodent samples. PeerJ., 4, e2216 10.7717/peerj.2216 27602258 PMC4991868

[mbo31105-bib-0023] Hänsch, S. , Cilli, E. , Catalano, G. , Gruppioni, G. , Bianucci, R. , Stenseth, N. C. , … Pallen, M. J. (2015). The *pla* gene, encoding plasminogen activator, is not specific to *Yersinia pestis* . BMC Research Notes, 535, 1–3. 10.1186/s13104-015-1525-x PMC4593223 26438258

[mbo31105-bib-0024] Hu, P. , Elliott, J. , McCready, P. , Skowronski, E. , Garnes, J. , Kobayashi, A. , … Garcia, E. (1998). Structural organization of virulence‐associated plasmids of *Yersinia pestis* . Journal of Bacteriology, 180, 5192–5202. 9748454 10.1128/jb.180.19.5192-5202.1998 PMC107557

[mbo31105-bib-0025] Iqbal, S. S. , Chambers, J. P. , Goode, M. T. , Valdes, J. J. , & Brubaker, R. R. (2000). Detection of *Yersinia pestis* by pesticin fluorogenic probe‐coupled PCR. Molecular and Cellular Probes, 14, 109–114. 10799272 10.1006/mcpr.2000.0295

[mbo31105-bib-0026] Iriarte, M. , & Cornelis, G. R. (1996). Molecular determinants of *Yersinia* pathogenesis. Microbiologia (Madrid, Spain), 12, 267–280. 8767710

[mbo31105-bib-0027] Isaacson, M. , Levy, D. , Pienaar, B. J. , Bubb, H. D. , Louw, J. A. , & Genis, G. K. (1973). Unusual cases of human plague in Southern Africa. South African Medical Journal, 47, 2109–2113. 4797079

[mbo31105-bib-0055] Liu, J. , Ochieng, C. , Wiersma, S. , Ströher, U. , Towner, J. S. , Whitmer, S. , … Fields, B. (2016). Development of a TaqMan array card for acute‐febrile‐illness outbreak investigation and surveillance of emerging pathogens, including ebola virus. Journal of Clinical Microbiology, 54, 49–58. 26491176 10.1128/JCM.02257-15 PMC4702733

[mbo31105-bib-0028] Livengood, J. , Hutchinson, M. L. , Thirumalapura, N. , & Tewari, D. (2020). Detection of Babesia, Borrelia, Anaplasma, and Rickettsia spp. in Adult Black‐Legged Ticks (Ixodes scapularis) from Pennsylvania, United States, with a Luminex Multiplex Bead Assay. Vector‐Borne and Zoonotic Diseases, 20, 406–411. 10.1089/vbz.2019.2551 31976829

[mbo31105-bib-0029] María, F. R. , Osikowicz, L. , Cáceres, A. G. , Luna‐Caipo, V. D. , Suarez‐Puyen, S. M. , Bai, Y. , & Kosoy, Y. (2019). Identification of *Bartonella rochalimae* in guinea pigs (*Cavia porcellus*) and fleas collected from rural peruvian households. American Journal of Tropical Medicine and Hygiene, 101, 1276–1281. 31674296 10.4269/ajtmh.19-0517 PMC6896888

[mbo31105-bib-0030] Matero, P. , Pasanen, T. , Laukkanen‐Ninios, R. , Tissari, P. , Tarkka, E. , Vaara, M. , & Skurnik, M. (2009). Real‐time multiplex PCR assay for detection of *Yersinia pestis* and *Yersinia pseudotuberculosis* . APMIS, 117, 34–44. 19161535 10.1111/j.1600-0463.2008.00013.x

[mbo31105-bib-0032] Moore, R. L. , & Brubaker, R. R. (1975). Hybridization of deoxyribonucleotide sequences of *Yersinia enterocolitica* and other selected members of *Enterobacteriaceae* . International Journal of Systematic Bacteriology, 25, 336–339.

[mbo31105-bib-0033] Neubauer, H. , Meyer, H. , Prior, J. , Aleksic, S. , Hensel, A. , & Splettstosser, W. (2000). A combination of different polymerase chain reaction (PCR) assays for the presumptive identification of *Yersinia pestis* . Journal of Veterinary Medicine, Series B, 47, 573–580. 11075545 10.1046/j.1439-0450.2000.00384.x

[mbo31105-bib-0034] Norkina, O. V. , Kulichenko, A. N. , Gintsburg, A. L. , Tuchkov, I. V. , Popov, Y. A. , Aksenov, M. U. , & Drosdov, I. G. (1994). Development of a diagnostic test for Yersinia pestis by the polymerase chain reaction. Journal of Applied Bacteriology, 76, 240–245. 8157543 10.1111/j.1365-2672.1994.tb01622.x

[mbo31105-bib-0035] Parkhill, J. , Wren, B. W. , Thomson, N. R. , Titball, R. W. , Holden, M. T. , Prentice, M. B. , … Barrell, B. G. (2001). Genome sequence of *Yersinia pestis*, the causative agent of plague. Nature, 413, 523–527. 11586360 10.1038/35097083

[mbo31105-bib-0036] Pawelczyk, O. , Asman, M. , & Solarz, K. (2019). The molecular detection of Anaplasma phagocytophilum and Rickettsia spp. in cat and dog fleas collected from companion animals. Folia Parasitologica, 66 10.14411/fp.2019.020 31823859

[mbo31105-bib-0037] Perry, R. D. , & Fetherston, J. D. (1997). *Yersinia pestis –* etiologic agent of plague. Clinical Microbiology Reviews, 10, 35–66. 8993858 10.1128/cmr.10.1.35 PMC172914

[mbo31105-bib-0038] Portnoy, D. A. , & Falkow, S. (1981). Virulence‐associated plasmids from *Yersinia enterocolitica* and *Yersinia pestis* . Journal of Bacteriology, 148, 877–883. 6273385 10.1128/jb.148.3.877-883.1981 PMC216287

[mbo31105-bib-0039] Quintard, K. , Dewitte, A. , Reboul, A. , Madec, E. , Bontemps‐Gallo, S. , Dondeyne, J. , … Sebbane, F. (2015). Evaluation of the role of the *opgGH* operon in *Yersinia pseudotuberculosis* and its deletion during the emergence of *Yersinia pestis* . Infection and Immunity, 83, 3638–3647. 26150539 10.1128/IAI.00482-15 PMC4534638

[mbo31105-bib-0040] Radnedge, L. , Gamez‐Chin, S. , McCready, P. M. , Worsham, P. L. , & Andersen, G. L. (2001). Identification of nucleotide sequences for the specific and rapid detection of *Yersinia pestis* . Applied and Environment Microbiology, 67, 3759–3762. 10.1128/AEM.67.8.3759-3762.2001 PMC93087 11472963

[mbo31105-bib-0041] Rajanna, C. , Revazishvili, T. , Rashid, M. H. , Chubinidze, S. , Bakanidze, L. , Tsanava, S. , … Sulakvelidze, A. (2010). Characterization of pPCP1 Plasmids in *Yersinia pestis* Strains Isolated from the Former Soviet Union. International Journal of Microbiology, 2010 10.1155/2010/760819 PMC3010648 21197443

[mbo31105-bib-0042] Randremanana, R. , Andrianaivoarimanana, V. , Nikolai, B. , Ramasindrazana, B. , Paireau, J. , Ten Bosch, Q. A. , … Rajerison, M. (2019). Epidemiological characteristics of an urban plague epidemic in Madagascar, August‐November, 2017: An outbreak report. The Lancet Infectious Diseases, 19, 537–545. 30930106 10.1016/S1473-3099(18)30730-8 PMC6483974

[mbo31105-bib-0043] Respicio‐Kingry, L. B. , Yockey, B. M. , Acayo, S. , Kaggwa, J. , Apangu, T. , Kugeler, K. J. , … Petersen, J. M. (2016). Two distinct *Yersinia pestis* populations causing plague among humans in the West Nile region of Uganda. PLoS Neglected Tropical Diseases, 10, e0004360. 26866815 10.1371/journal.pntd.0004360 PMC4750964

[mbo31105-bib-0044] Riehm, J. M. , Rahalison, L. , Scholz, H. C. , Thoma, B. , Pfeffer, M. , Razanakoto, L. M. , … Tomaso, H. (2011). Detection of *Yersinia pestis* using real‐time PCR in patients with suspected bubonic plague. Molecular and Cellular Probes, 25, 8–12. 20933595 10.1016/j.mcp.2010.09.002

[mbo31105-bib-0045] Shi, L. , Yang, G. , Zhang, Z. , Xia, L. , Liang, Y. , Tan, H. , … Wang, P. (2018). Reemergence of human plague in Yunnan, China in 2016. PLoS One, 13, e0198067. 29897940 10.1371/journal.pone.0198067 PMC5999221

[mbo31105-bib-0046] Stenseth, N. C. , Atshabar, B. B. , Begon, M. , Belmain, S. R. , Bertherat, E. , Carniel, E. , … Rahalison, L. (2008). Plague: past, present, and future. PLoS Med, 5, e3. 10.1371/journal.pmed.0050003 PMC2194748 18198939

[mbo31105-bib-0047] Stewart, A. , Satterfield, B. , Cohen, M. , O'Neill, K. , & Robison, R. (2008). A quadruplex real‐time PCR assay for the detection of *Yersinia pestis* and its plasmids. Journal of Medical Microbiology, 57, 324–331. 10.1099/jmm.0.47485-0 18287295

[mbo31105-bib-0048] Tomaso, H. , Reisinger, E. C. , Al, D. S. , Frangoulidis, D. , Rakin, A. , Landt, O. , & Neubauer, H. (2003). Rapid detection of *Yersinia pestis* with multiplex real‐time PCR assays using fluorescent hybridisation probes. FEMS Immunology and Medical Microbiology, 38, 117–126. 13129646 10.1016/S0928-8244(03)00184-6

[mbo31105-bib-0049] Tripp, D. W. , Gage, K. L. , Montenieri, J. A. , & Antolin, M. F. (2009). Flea abundance on black‐tailed prairie dogs (*Cynomys ludovicianus*) increases during plague epizootics. Vector‐Borne and Zoonotic Diseases, 9, 313–321. 19492944 10.1089/vbz.2008.0194

[mbo31105-bib-0051] Williams, J. E. , Harrison, D. N. , & Cavanaugh, D. C. (1975). Letter: Cryptic infection of rats with non‐encapsulated variants of *Yersinia pestis* . Transactions of the Royal Society of Tropical Medicine and Hygiene, 69, 171–172. 1145712 10.1016/0035-9203(75)90039-5

[mbo31105-bib-0052] Williams, J. E. , Harrison, D. N. , Quan, T. J. , Mullins, J. L. , Barnes, A. M. , & Cavanaugh, D. C. (1978). Atypical plague bacilli isolated from rodents, fleas, and man. American Journal of Public Health, 68, 262–264. 637172 10.2105/ajph.68.3.262 PMC1653920

[mbo31105-bib-0053] Winter, C. C. , Cherry, W. B. , & Moody, M. D. (1960). An unusual strain of Pasteurella pestis isolated from a fatal human case of plague. Bulletin of the World Health Organization, 23, 408–409. 13845309 PMC2555592

[mbo31105-bib-0054] Woron, A. M. , Nazarian, E. J. , Egan, C. , McDonough, K. A. , Cirino, N. M. , Limberger, R. J. , & Musser, K. A. (2006). Development and evaluation of a 4‐target multiplex real‐time polymerase chain reaction assay for the detection and characterization of *Yersinia pestis* . Diagnostic Microbiology and Infectious Disease, 56, 261–268. 16949784 10.1016/j.diagmicrobio.2006.06.009

